# Stability of Thermoablation Antenna Using a Patient‐Mounted Navigation System: Initial Clinical Experience

**DOI:** 10.1111/1754-9485.13840

**Published:** 2025-02-06

**Authors:** Mohammed Shamseldin, Herbert Sayer, Ralf Puls

**Affiliations:** ^1^ Department of Radiology Helios Klinikum Erfurt Thuringia Germany; ^2^ Department of Hematology and Oncology Helios Klinikum Erfurt Thuringia Germany

**Keywords:** Cube Navigation System, euclidian distance, microwave ablation

## Abstract

**Purpose:**

CT‐guided microwave ablation (MWA) has become a standard procedure for a range of therapeutic and diagnostic indications, but accurate and stable positioning of the antenna is critical. In this retrospective case series, a navigation guide with a physical cube component, the Access Cube (AC), was investigated as a stability support in addition to its use as a navigation system. To our knowledge, this is the first investigation of stability in MWA.

**Materials and Methods:**

Eight MWAs performed at one centre using the AC were reviewed for clinical and technical success. The stability of the antenna was assessed by subjectively comparing the Euclidian distance (ED) between the needle tip location in the final control scan and confirmation scan. A practising radiologist not associated with the study independently assessed the coordinates, and the mean was calculated from the results.

**Results:**

Six patients (eight procedures) were included (4 females). Mean age of the patients was 75.8 years (range 58–87). Diagnoses included liver metastasis (4, 50%), renal cell carcinoma (2, 25%) and 1 case each (12.5%) of hepatocellular carcinoma and lung metastasis. Mean tumour size was 2.4 cm (range 1.0–4.3 cm), with a mean depth of 10.6 cm (range 5–18 cm). Mean ED of needle tip between final control scan and confirmation scan was 5.82 mm. Technical and clinical success were achieved in all cases with one Grade 2 complication arising.

**Conclusion:**

Usage of the AC was a beneficial addition to the MWA process. Good stability of the antenna was achieved when placed through the AC, eliminating the need for the clinician to manually hold the antenna in place during ablation.

**Level of Evidence:**

Level 4, Case Series.

## 1 Introduction

Microwave ablation (MWA), a commonly used percutaneous imaging‐guided intervention for tumour treatment, has evolved into a standard procedure for a wide range of therapeutic and diagnostic indications [1, 2, 3, 4]. The success of MWA procedures hinges on accurate and stable positioning of the antenna. Inaccurate placement or insufficient ablation may lead to prolonged treatment times, unnecessary tissue damage, adverse events or even treatment failures [5].

Not only is the initial placement of the antenna important, but also the ablation antenna should firmly stay in place after the introduction. However, the heavy handle of the ablation antenna and cable has the potential to shift the angle and position of the antenna, even with the antenna fully embedded in tissue. When the lesion is superficial, this problem is only exacerbated.

To combat this, standard practice entails the physician holding the antenna during the ablation or relying on soft tissue to provide stability for the ablation antenna during ablations and control/interim scans. It must be noted that this is not an option during control/interim scans due to radiation exposure to the operator. As there is a considerable risk of the ablation antenna moving after the initial placement, many centres utilise thermometric software capable of estimating the extent of the ablation zone, thus allowing compensation for movement of the ablation antenna after placement. Alternatively, confirmation is also commonly performed via the conventional side‐by‐side visual assessment, which has been shown to have an error rate of up to 44% [6].

With relation to accuracy of placement, computed tomography (CT) is often utilised for image guidance during MWA procedures owing to its good availability, high spatial resolution, rapid volume acquisition, ability to capture detailed images of a variety of body tissue types and comparatively low cost [5, 7, 8]. This makes CT an advantageous choice in guiding these procedures effectively and economically. In most centres, needle placement using CT is typically done manually, specifically through the free‐hand method (FHM). Using this approach, the physician approximates the translation from the planned to the actual needle insertion angle. The accuracy of this method relies heavily on the experience, visual and spatial abilities of the physician. While the FHM may be suitable for basic in‐plane interventions, when an out‐of‐plane trajectory is necessary, some access routes can be difficult to estimate. A variety of CT navigation systems have been introduced to address this issue, including systems based on optical and electromagnetic inputs [7]. Nevertheless, the widespread adoption of these systems has been constrained by various factors such as cost, extended procedural time and heightened workflow complexity.

**FIGURE 1:**
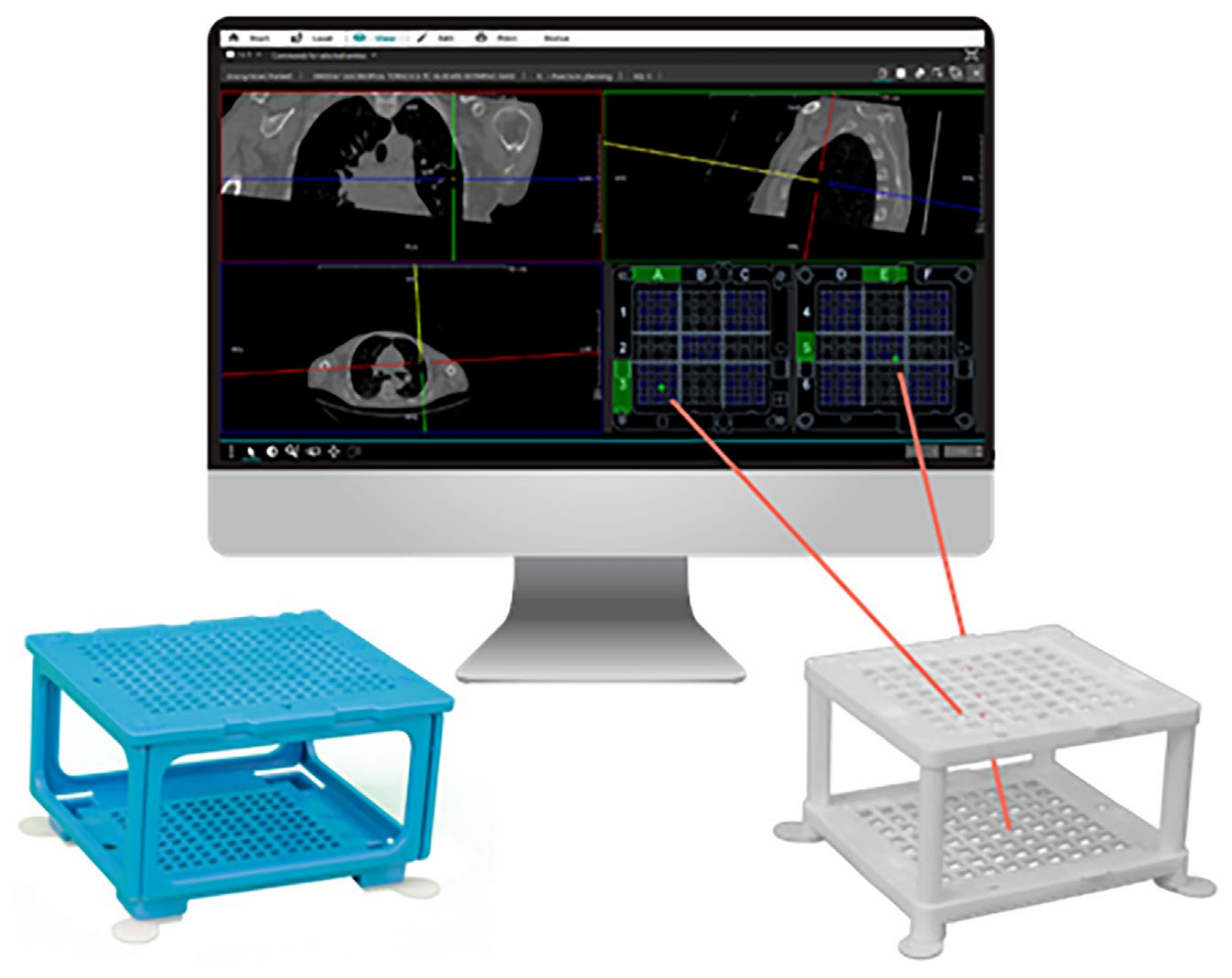
The Cube Navigation System comprising software for planning the puncture route using a virtual needle and cube accessories (left, Puncture Cube for 18–22G instruments; right, Access Cube for 10–20G instruments). The route through the cube is displayed in the lower right corner, which corresponds to the top and bottom frames of the cube. Image used with permission from Medical Templates AG.

A new needle guidance system, the ‘Cube Navigation System’ (CNS; Medical Templates AG, Egg, Switzerland), was recently introduced (Figure 1). The CNS comprises a small, patient‐mounted, self‐adhesive navigation cube with multiple through holes in the upper and lower plate and accompanying software that identifies the cube in the planning scan enabling the physician to plan an optimal trajectory to the target using a virtual needle. The route is transferred to coordinates on both the top and bottom grid of the cube. The physical needle is then inserted into the respective holes on the cube. Due to its shape and adhesive pads, the navigation cube can act as support for the ablation antenna during placement (i.e., when the needle is only partially inserted) as well as once the navigation has been completed, although this has not been previously investigated.

The CNS is a simple yet accurate way to guide needle punctures, as shown by multiple in vitro phantom studies [9, 10, 11] and two clinical case series [12, 13]. The studies have shown punctures with the CNS to be faster and more accurate compared to the conventionally used free‐hand method.

In this retrospective case series, we describe our initial clinical experiences using the CNS for navigation and stability of the ablation antenna. For this study, the navigation cube variant Access Cube (AC) was used. The system was applied in a small series of eight patients referred for MWA of tumours in the lung, liver and kidney.

## 2 Methods

### 2.1 Patients

We retrospectively selected from our database all cases where the CNS had been used for a percutaneous thermal ablation procedure and where an out‐of‐plane approach was required to obtain access between July 2023 and February 2024. Indication for MWA was based on internal multidisciplinary tumour board recommendations of non‐resectable tumours or patients not fit for surgery. Ahead of treatment, all patients were screened for coagulation disorders, oral anticoagulation, platelet‐inhibiting medication and allergies. Only monotherapy with NSAID was allowed. In cases where the application of contrast medium to improve visualisation of the lesions was mandatory, kidney function and thyroid gland hormones were also required. Before MWA planning, previous MRI or CT scans were reviewed. If findings were consistent with the diagnosis, a preliminary access route as well as the patient's position during the ablation process were planned. All procedures were performed under general anaesthesia.

All patients were informed about the technique and potential complications of MWA.

Clinical success was assessed based on the complete ablation of the target lesion in the best imaging modality to visualise the lesion.

The summary of all patients and ablation cases performed showing the diagnosis, stability of the needle, total procedural time, number of scans and the radiation dose of each patient is shown in Table 1.

**TABLE 1:** Summary of results.

Participant no.	Procedure no.	Patient age/sex	Diagnosis	ED (mm)	Total time (min)	Number of scans	Radiation dose
1	1	84/F	Liver metastasis (colon)	7.64	38	5	623
1	2	84/F	Lung metastasis (colon)	3.62	25	3	270
2	3	72/F	Hepatocellular carcinoma	8.83	51	4	236.7
3	4	87/F	Renal cell carcinoma	5.86	26	5	606
1	5	84/F	Liver metastasis (colon)	5.11	49	10	1530
4	6	72/F	Liver metastasis (rectum)	6.11	36	3	1420
5	7	82/M	Renal cell carcinoma	3.09	58	7	2734
6	8	58/M	Liver metastasis (colon)	6.31	72	8	1871

### 2.2 CNS and AC

The AC is a newly developed navigation cube for the CNS (Medical Templates AG, Egg, Switzerland). It is comprised of two grids held parallel within a frame at a 50 mm distance, each with 72 alphanumerically marked through‐holes that fit needles from 20 to 10G. Each hole has four corners in which the needle can rest, allowing for precise alignment and additional stability once the instrument has been inserted. The bottom template is supported by four self‐adhesive feet, each measuring 9.6 mm in height, designed to adapt to the contours of the skin. Both the templates and the frame are fully detachable even with the needle in position, enabling complete access to the intervention area. This facilitates slight adjustments to the needle orientation post‐placement if necessary.

The sterile AC is positioned over the intended intervention site after disinfecting the site and covering the surroundings with surgical drapes. Following that, a planning scan is performed. The software identifies the AC and presents a modified 3D multiplanar reconstruction (MPR) view, incorporating a ‘virtual needle’ that can be freely adjusted for puncture planning. Real‐time coordinates that correspond to the templates are displayed by the software. Upon selecting a trajectory, the physical needle is inserted through the designated holes and corners of the AC. Once in position, the AC serves as a needle support, aiding in maintaining the correct angle. Depth control is achieved through control scans or by utilising the distance markers presented on the virtual needle.

Synedra View Professional 23.0.0, which is necessary to plan the puncture route supports the following operating systems: Windows 10 32‐bit and 64‐bit (on Intel/AMD processors), Windows 11 (on Intel/AMD processors), Windows Server 2016 with Citrix Virtual Apps and Desktops 1912 LTSR (or later) as well as 12 Monterey or 13 Ventura mac operating systems. The minimum and recommended system requirements to run Synedra View Professional 23.0.0 on diagnostic and viewing workstations are mentioned in detail in Tables 2 and 3.

**TABLE 2:** Hardware requirements Synedra View Professional diagnostic workstations.

	Minimum requirement	Recommendation
Processor	4 cores	12 cores
RAM	16 GB	32 GB of (on 64‐bit operating systems)
Hard drives	250 GB SATA or more	250 GB SSD or more
Graphics card	As a rule, graphics cards could be supplied for the diagnostic monitors—if necessary—and have been specifically adapted to these	
CPU support	Minimum requirement is a graphics card that supports OpenCL 1.2 and grey‐scales images via OperaCL as well as a 64‐bit operating system	
Network connection	Gigabit Ethernet	
Monitors	Diagnostic monitors compliant with normative requirements Control monitors: TFT 1280*1024 resolution or more	
Power supply unit	550 W minimum	
Further requirements	Make sure that cooling and the power supply are sufficiently dimensioned to run the diagnostic graphics card. Furthermore, only use the specifically adapted graphics card driver that comes with the diagnostic graphics card	

**TABLE 3:** Hardware requirements Synedra View Professional viewing workstations.

	Minimum requirement	Recommendation
Processor	4 cores	8 cores
RAM	8 GB	16 GB of (on 64‐bit operating systems)
Hard drives	250 GB SATA or more	250 GB SSD or more
Network connection	100 MBit full duplex	Gigabit Ethernet
Monitors	TFTs 400:1 contrast ratio or more, 1280*1024 resolution or more	Monitors with DICOM LUT

### 2.3 Procedure Description

**FIGURE 2:**
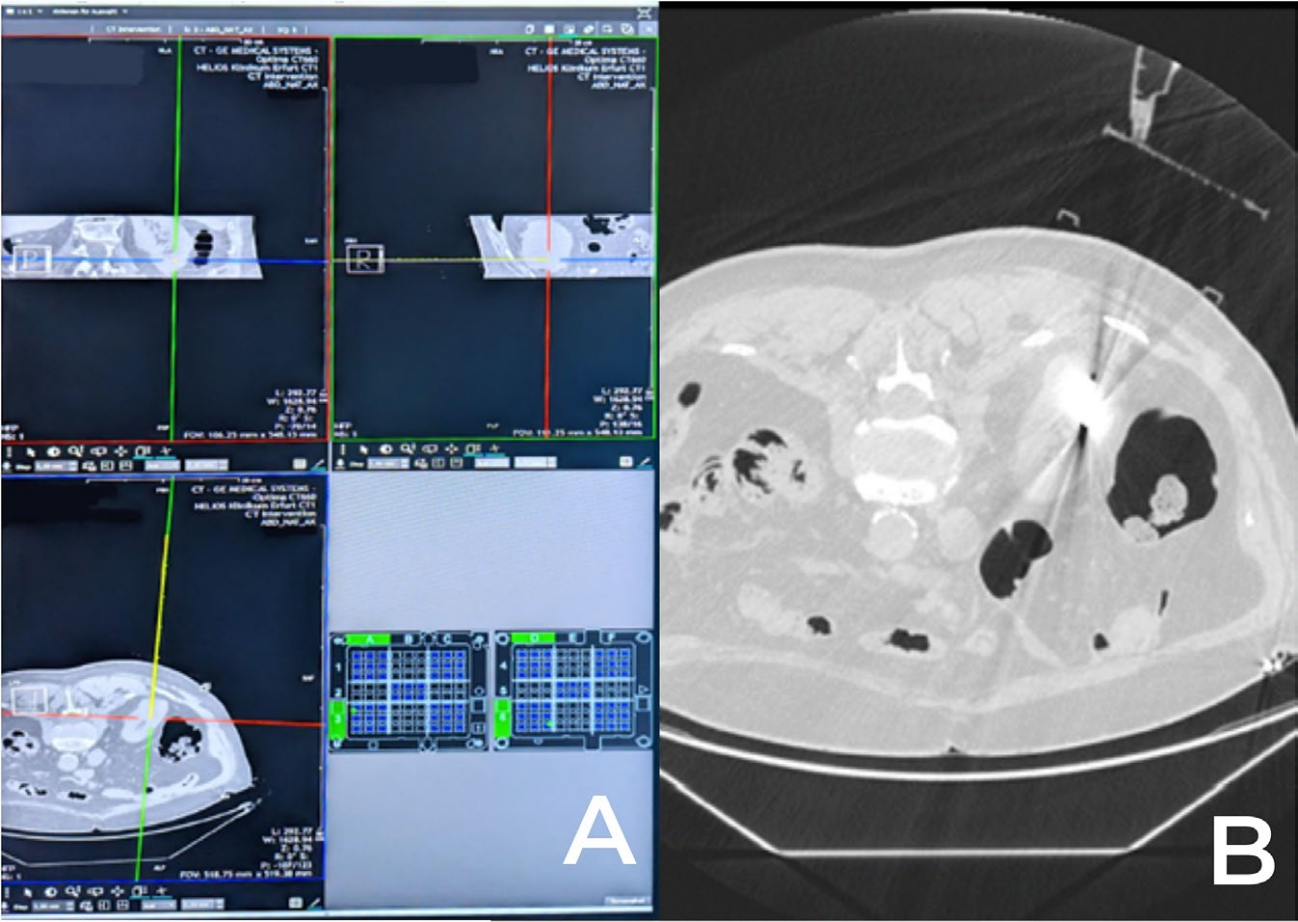
Navigation for a renal cell carcinoma MWA. Section A shows the navigation in the CNS, with the yellow line indicating a virtual needle. Section B shows the interim scan with the AC adhered to the patient and the ablation antenna inserted into place.

**FIGURE 3:**
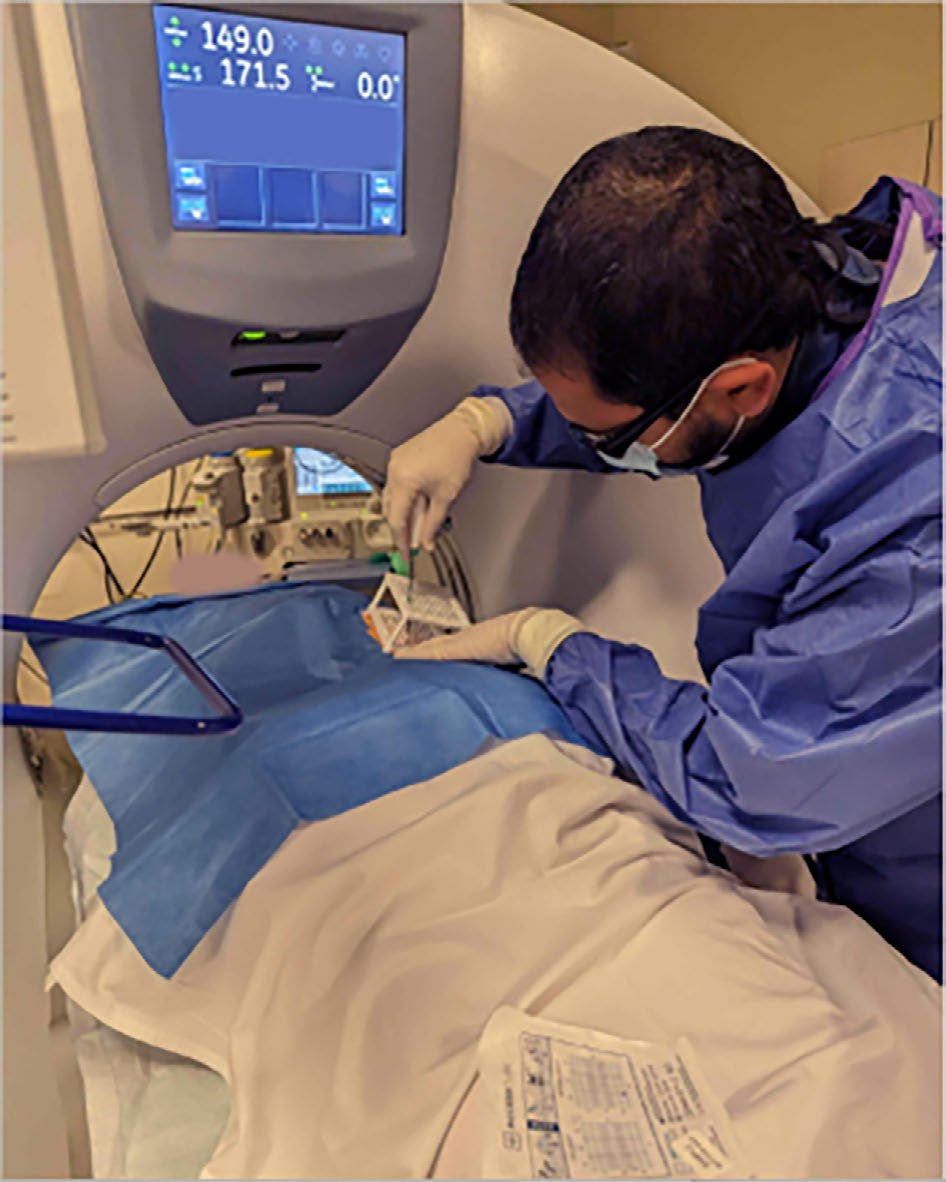
Using the coordinates from the CNS software (pictured here transferred to the grid on the label of the AC, bottom centre), the ablation antenna was placed into the patient and advanced towards the target.

**FIGURE 4:**
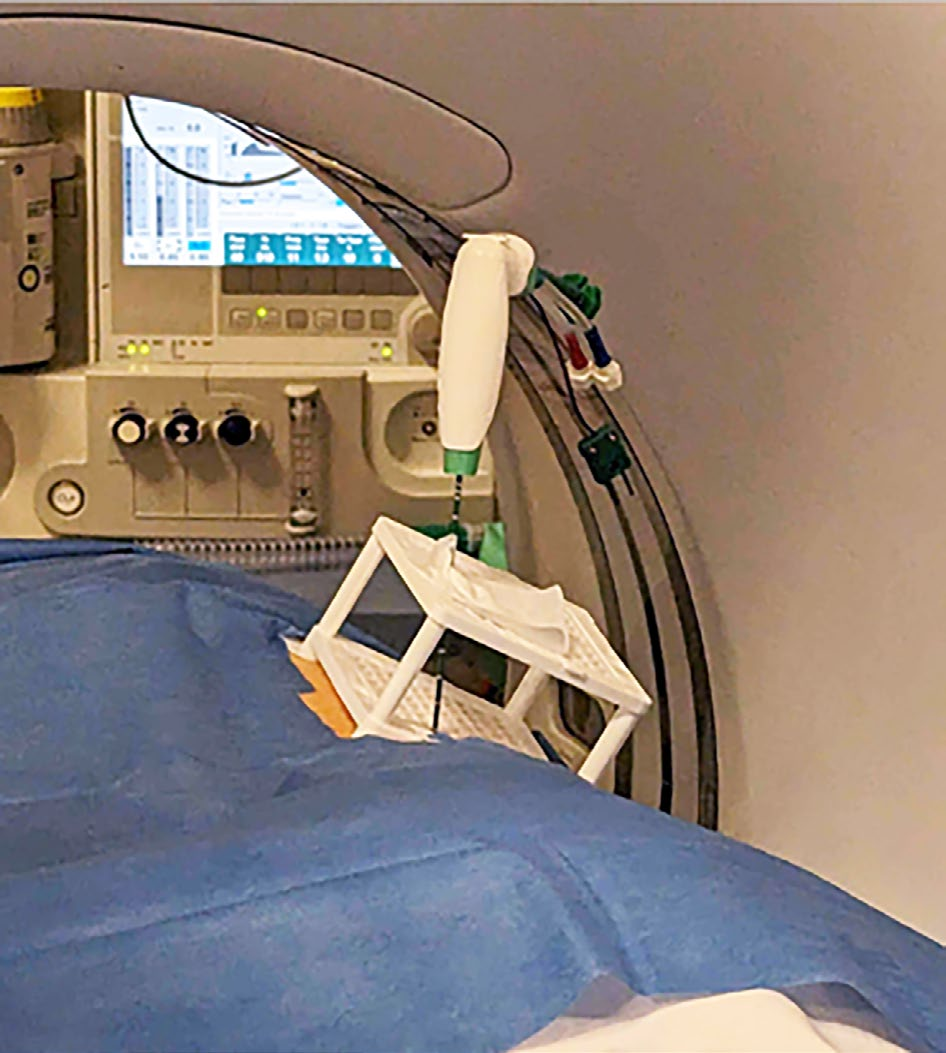
The AC remained assembled after placement on the patient and acted as a needle holder for the ablation antenna during the MWA procedure.

Patients were placed on the CT table in either a prone or supine position, depending on procedural planning. Ahead of the MWA, all patients had an intravenous access placed. Vital signs were monitored throughout the procedure, including oxygen saturations and ECG. CT guidance with sequential scanning (120 Kv, 240 mAs tube current and 0.625 mm slice thickness) was used for planning, targeting and intraprocedural modification. After skin disinfection, the AC was placed over the approximate puncture site and a CT planning scan was obtained. The images were transferred into the software, and the access route to the target was determined. The coordinates to introduce the antenna were recorded for the upper and lower plate (Figure 2). A 16G MWA antenna (HS Hospital Service, Rome, Italy) was introduced (length 15–20 cm) and its approach to the target was evaluated with sequential CT scans. All ablations were performed with Amica Microwave ablation machines (HS Hospital Service, Rome, Italy) (Figure 3). Although the AC can be dismantled in these procedures, it was left assembled on the patient as a support for the ablation antenna (Figure 4). The ablation session was performed with an ablation protocol according to the manufacturer's guidelines. After the ablation was performed, the single‐use AC was discarded with the regular hospital waste. Patients remained in the hospital overnight before discharge. All eight procedures were performed by a highly experienced interventional radiologist with over 10 years of experience in percutaneous ablation techniques but had no prior experience with the CNS.

Stability of the ablation antenna was calculated by evaluating the antenna tip location in the final intraprocedural scan and antenna tip location in the confirmation scan (after the ablation procedure). To reduce bias, evaluation of the needle tip location was performed by a practising radiologist not associated with the study. From this, the Euclidean distance (ED), defined as the length of the line segment between two points in the 3D Euclidean space, was calculated. The reason for using the ED as an indicator is to provide a 3D evaluation of the stability of the antenna tip during the ablation procedure, which is crucial to ensure a successful and complete ablation of the target lesion. Any dislocation of the needle in any dimension might cause serious complications such a thermal injury of the neighbouring organs or incomplete ablation of the target lesion. ED between the antenna tip in the final intraprocedural scan and confirmation scan, was calculated using the 3D coordinates (x y z) and the following formula:

$$\mathrm{ED}={\left({\mathrm{Dx}}_{\text{control}}-{\mathrm{Dx}}_{\text{conf}}\right)}^{2}+{\left({\mathrm{Dy}}_{\text{control}}-{\mathrm{Dy}}_{\text{conf}}\right)}^{2}+{\left({\mathrm{Dz}}_{\text{control}}-{\mathrm{Dz}}_{\text{conf}}\right)}^{2}$$

Additionally, procedure duration (defined as time from the first CT topogram to the last CT control based on the timestamps of the CT scans), number of corrections of the needle path, number of scans, radiation dose measured in mGy/cm taken from the patient protocol and technical success were retrospectively evaluated.

### 2.4 Safety

Patient records were screened for complications up to 30 days post‐procedure. Complications were graded according to the CIRSE Classification System for Complications [14].

### 2.5 Statistics

Descriptive statistics were used to summarise the data. No statistical analysis was performed, given the small sample size.

## 3 Results

### 3.1 Patient Inclusion

A total of six patients (eight procedures) were included in the analysis. The procedure was repeated on one patient due to an incomplete primary ablation, and the same patient initially underwent a lung and liver ablation on the same day, which was counted as two procedures. Patient demographics are presented in Table 4. Patients had a mean age of 75.8 years (range 58–87), with four female and two male patients. The most common diagnosis was liver metastasis (four procedures, 50%), renal cell carcinoma (RCC) (two procedures, 25%), and finally, 1 case each (12.5%) of hepatocellular carcinoma (HCC) and lung metastasis. The mean tumour size was 2.4 cm (range 1.0–4.3 cm), with a mean depth of 10.6 cm (range 5–18 cm).

**TABLE 4:** Patient demographics.

Demographics	*n*	%
Patients	6	
Procedures	8	
Sex (number F, % F)	4	70
Mean age (Years, Range)	75.8	72–87
Tumour type	Liver metastasis (4)	50
	Renal cell carcinoma (2)	25
	HCC (1)	12.5
	Lung metastasis (1)	12.5
Tumour size (mean, range)	2.4 cm	4.3–1.0 cm
Tumour depth (mean, range)	10.6 cm	5–18 cm

### 3.2 Antenna Stability

Mean ED of the antenna was 5.82 mm (range 8.83–3.09 mm).

### 3.3 Procedure Results

Technical success defined as the ability to reach the destination point and achieve reasonable stability of the ablation antenna was achieved in all cases. An ED between the antenna tip in the final intraprocedural scan and confirmation scan of less than 1 cm was considered reasonably stable. The mean planning time was 7.1 min with a range of 2–15 min. The mean number of control scans was 6 (range 3–10). Two procedures required needle corrections during placement, in each case, two corrections were performed.

Clinical success was achieved in 7 of the 8 procedures (88%) with complete ablation of the lesions achieved. In one case, a repetition of the procedure due to poor visualisation of the lesion was necessary.

Mean radiation dose area product for the entire procedure was 1197 mGy/cm (range 270–2734).

Complications: A Grade 2 complication of participant number 6 with a subcapsular bleeding was identified on the standard post‐procedural CT‐scan on the following day, which was successfully treated conservatively without any necessary intervention.

## 4 Discussion

Usage of the AC was, in this initial case series, a beneficial addition to the MWA process. Good stability of the antenna was achieved when placed through the AC, eliminating the need for the clinician to manually hold the antenna in place during ablation. However, as the stability of the ablation antenna has not previously been studied, we are unable to compare it with other rates in the literature.

Lateral access routes were possible with the CNS, allowing the insertion of the needle in a one‐step procedure, allowing an easier flow of the table through the gantry and a more stable position of the needle with a low risk of dislocation. In most cases, an additional fixation of the CNS using external sterile skin plaster to stabilise the AC was necessary to avoid any possible movements of the CNS under the weight of the ablation antenna as well as the repeated movement of the CT table during the procedure.

The procedure was repeated on one patient secondary to incomplete primary ablation of a liver lesion (Patient 1, Procedures 1 and 5). Due to the poor visibility of the lesion in CT during the first procedure, placing the needle centrally through the lesion was challenging. So despite the needle being placed as intended, the initial positioning of the needle was not ideal leading to incomplete ablation. In the second attempt, the primary ablation zone was used to successfully position the needle centrally through the tumour residue.

Thus, in our experience, the CNS brings substantial value to MWA procedures both as a navigational aid as well as in a needle holder capacity.

## 5 Limitations

As a small, retrospective case series, generalisability is limited. Other studies may evaluate the stability of ablation antennas with the CNS against a control group using a prospective design. Additionally, the interventions were performed by a single clinician, which may have impacted the results.

## 6 Conclusion

The CNS enabled precise calculation of a double‐oblique access route, allowing the user to select an optimal pathway, and appeared to minimise variability in procedure time as already described in previous reports [12, 13]. In this case series, MWAs with the AC were found to be stable, accurate and streamlined which is the novelty of this study in additionally assessing the stability of the needle when using the AC. A more comprehensive assessment of the CNS, including prospective and comparative studies with larger patient samples encompassing various indications and procedures is recommended.

## Author Contributions

M.S. was responsible for performing the procedures, data gathering, statistical analysis and was the main contributor in writing the manuscript. H.S. was responsible for the interdisciplinary tumour board decisions. R.P. was responsible for the supervision of the thermoablation interventions. All authors read and approved the final manuscript.

## Ethics Statement

The authors have nothing to report.

## Consent

The authors have nothing to report.

## Conflicts of Interest

The authors declare no conflicts of interest.

## Data Availability

Internal PACS system of Helios Klinikum Erfurt.
